# Effects of Roxithromycin Exposure on the Nitrogen Metabolism and Environmental Bacterial Recruitment of *Chlorella pyrenoidosa*

**DOI:** 10.3390/plants14172774

**Published:** 2025-09-04

**Authors:** Jiping Li, Ying Wang, Zijie Xu, Chenyang Wu, Zixin Zhu, Xingsheng Lyu, Jingjing Li, Xingru Zhang, Yan Wang, Yuming Luo, Wei Li

**Affiliations:** 1 School of Life Sciences, Huaiyin Normal University, Huai’an 223300, China; jipingli@hytc.edu.cn (J.L.); wy02071109@163.com (Y.W.); zijiexu2005@163.com (Z.X.); chenyangWu0713@163.com (C.W.); zixinzhu1217@163.com (Z.Z.); lxs04162025@163.com (X.L.); jingjingli0527@163.com (J.L.); ZXR1906230088@163.com (X.Z.); yanwang@hytc.edu.cn (Y.W.); yumingluo@163.com (Y.L.); 2 Jiangsu Collaborative Innovation Center of Regional Modern Agriculture & Environmental Protection, Huaiyin Normal University, Huai’an 223300, China; 3 College of Ecology and Environment, Nanjing Forestry University, Longpan Road 159, Nanjing 210037, China

**Keywords:** microalgae, macrolides, phycospheric bacteria, antibiotic stress, denitrification

## Abstract

The ecotoxicity induced by macrolides has attracted widespread attention, but their impacts on the nitrogen metabolism and symbiotic environmental bacteria of microalgae remain unclear. This study examined the effects of roxithromycin (ROX) on the growth, chlorophyll levels, and nitrogen metabolism of *Chlorella pyrenoidosa*; investigated the changes in the composition and functions of environmental bacterial communities; and finally, analyzed the relationship between microalgae and environmental bacteria. The results indicated that all concentrations of ROX (0.1, 0.25, and 1 mg/L) inhibited microalgae growth, but the inhibition rates gradually decreased after a certain exposure period. For instance, the inhibition rate in the 1 mg/L treatment group reached the highest value of 43.43% at 7 d, which then decreased to 18.93% at 21 d. Although the total chlorophyll content was slightly inhibited by 1 mg/L ROX, the Chl-a/Chl-b value increased between 3 and 21 d. The nitrate reductase activities in the three treatments were inhibited at 3 d, but gradually returned to normal levels and even exceeded that of the control group at 21 d. Under ROX treatment, the consumption of NO_3_^−^ by microalgae corresponded to the nitrate reductase activity, with slower consumption in the early stage and no obvious difference from the control group in the later stage. Overall, the diversity of environmental bacteria did not undergo significant changes, but the abundance of some specific bacteria increased, such as nitrogen-fixing bacteria (*unclassified-f-Rhizobiaceae* and *Mesorhizobium*) and organic contaminant-degrading bacteria (*Limnobacter*, *Sphingopyxis*, and *Aquimonas*). The 0.25 and 1 mg/L ROX treatments significantly enhanced the carbohydrate metabolism, cofactor and vitamin metabolism, amino acid metabolism, and energy metabolism of the environmental bacteria, but significantly downregulated nitrogen denitrification. This study provides new insights into the environmental bacteria-driven recovery mechanism of microalgae under antibiotic stress.

## 1. Introduction

Macrolide antibiotics are widely used in the treatment and prevention of respiratory infections, urinary infections, and alimentary infections. The World Health Organization considers macrolides to be some of the highest priority drugs due to their broad spectrum effects and effectiveness [1,2]. Population explosions and advances in pharmaceutical technology have led to increases in the use of antibiotics. In 2023, the defined daily dose per 1000 inhabitants of macrolides was 835 in the United States and 1017 in India [3]. The annual use of antibiotics in China was 162,000 t, with macrolides being the most used, accounting for 26.05% [4]. Some unmetabolized and unused macrolides ultimately enter surface waters, leading to high concentrations and detection frequencies. For instance, the highest concentration of roxithromycin (ROX) was 310.90 ng/L in the upper region of the Yangtze River, China, with a detection frequency of 96.70% [5]. The maximum concentration of erythromycin reached 1149 ng/L in the rivers of Kumasi, Ghana [6]. This environmental issue is more severe in densely populated developing countries or regions [7,8]. Due to the high antibacterial efficacy of macrolide antibiotics, the ecological toxicity effects they cause to non-target organisms in aquatic systems have garnered widespread attention.

Macrolides exposure has been demonstrated to exhibit toxic effects on aquatic organisms across different trophic levels [9,10,11]. In particular, microalgae, due to their high sensitivity to macrolides and role as primary producers in aquatic ecosystem, have attracted more attention [12,13]. The growth of microalgae cells is progressively inhibited as the concentration of macrolides, such as erythromycin, azithromycin, and ROX, increases [14,15,16]. On the one hand, the photosynthetic efficiency of microalgae is closely related to their carbon fixation ability and growth, with the concentration of various pigments in cells determining the photochemical conversion efficiency. Macrolides inhibit the expression of genes involved in the biosynthesis of pigments, leading to a decrease in the production of photosynthetic pigments on thylakoid membranes [17,18]. For example, 5–100 μg/L azithromycin and 100 μg/L erythromycin decreased the chlorophyll-a (Chl-a), Chl-b, and carotenoid contents; these photosynthetic pigments are generally considered the most important components for harvesting light [13,19]. Macrolide exposure may also interfere with electron transport processes on thylakoid membranes, thereby affecting the conversion of ADP to ATP [20]. A deeper explanation is that the expression of genes associated with these processes in the photosynthetic system is downregulated. These genes include *chlH* and *bchM*, which are involved in porphyrin and chlorophyll metabolism [18]; *psaA* and *psbA*, which are involved in photosystems I and II [17]; and *atpA* and *atpB*, which are related to chloroplast ATP synthase [21]. On the other hand, the disturbed electron transport processes induce the accumulation of electrons; these excess electrons result in the generation of reactive oxygen species [22]. The presence of reactive oxygen species can induce lipid peroxidation of membranes, and this damage to organelle membranes inevitably impairs their cytological functions, resulting in a decrease in various metabolic activities within the cell and ultimately leading to reduced cell proliferation of the microalga [23]. Most of the previous studies have focused on the effects of macrolide exposure on microalgae’s photosynthetic efficiency, carbon metabolism, and antioxidant enzyme responses, with few studies addressing the impact on microalgae’s nitrogen metabolism. Since nitrogen absorption and transformation are also crucial for microalgae growth, it is essential to investigate the impact of macrolides on nitrogen metabolism.

Microalgae in the aquatic environment recruit bacteria to colonize their surrounding microenvironment, forming microalgae–bacteria consortia. These bacteria are commonly referred to as symbiotic environmental bacteria [24]. Microalgae and environmental bacteria exchange nutrients, metabolites, and infochemicals. This symbiotic relationship enables them to establish a relatively stable consortium in response to environmental changes [25,26]. Due to the antimicrobial properties of antibiotics, their presence in aquatic environments is bound to affect the relationship between microalgae and environmental bacteria. Many previous studies indicated that the presence of environmental bacteria may enhance the adaptability of microalgae to antibiotic stress. For example, tetracycline and ciprofloxacin exposure drove selective enrichment of multiple probiotic bacterial taxa within the symbiotic microenvironment of microalgae through niche modifications [27]. Tetracycline and sulfadiazine exposure altered the metabolism and exudates of microalgae, with biomacromolecules such as lysoPC, methionine, and sulfatide modulating the bacterial community assembly in the phycosphere, thereby mediating the distribution and abundance of antibiotic resistance genes [26]. Microalgae-recruited bacterial consortia affect microalgal growth under environmental stress through various ecological mechanisms: biotransformation of exogenous pollutants such as antibiotics or heavy metals via specialized catabolic pathways [26]; modulation of nitrogen cycling through metabolic coupling between microalgae and recruited bacteria [28,29]; and enhancement of microalgae stress acclimation via inter-species signal transduction [27,30]. Consequently, antibiotic exposure perturbs the symbiotic relationship of microalgae–bacteria consortia, and changes in the composition and function of environmental bacteria may affect the nitrogen metabolism and growth of microalgae. Our previous investigation identified various environmental bacteria in an open microalgae culture system where *Chlorella pyrenoidosa* (*C. pyrenoidosa*) was exposed to ROX. These environmental bacteria were recruited by microalgae under non-sterile culture conditions [28,31]. However, the functional roles of these bacteria in mediating microalgal acclimation to antibiotic stress remain poorly characterized. This critical knowledge gap should be addressed to accurately assess the ecotoxicological impacts of antibiotics in aquatic environments.

In summary, this study employed ROX, a frequently detected macrolide antibiotic as the exogenous stressor, and *C. pyrenoidosa*, a model freshwater primary producer with high ecotoxicological sensitivity as the test organism, to systematically achieve the following research objectives: (1) to investigate the chronic toxic effects of ROX on the growth and chlorophyll content of microalgae; (2) to investigate the nitrate reductase (NR) activity of microalgae and the nitrate conversion activity in the symbiotic system; and (3) to investigate the changes in the composition and function of environmental bacteria.

Based on these research aims, this study is the first to perform a coupled analysis of the nitrate reductase activity in microalgae cells and the nitrogen metabolism of environmental bacteria when exposed to roxithromycin. It also demonstrates that the reduced inhibition rate under high-concentration ROX exposure is linked to environmental bacterial-driven mechanisms. Moreover, this research provides a conceptual model of “microalgae–bacteria collaborative resistance” that can be used by further studies on the ecological toxicity of antibiotics and microalgae-based wastewater treatment. The discussion on the interrelationship between environmental bacteria and microalgae metabolism will provide a deeper understanding of the aquatic toxicity of macrolide antibiotics.

## 2. Materials and Methods

### 2.1. Reagents and Materials

ROX with a purity greater than 98% was purchased from Beijing J&K Scientific Ltd. (Beijing, China). The test organism *C. pyrenoidosa* (No. FACHB-11) was purchased from the Freshwater Algae Culture Collection at the Institute of Hydrobiology (Wuhan, China). The reagents used to make the BG11 culture medium are shown in Table S1 (Supplementary Materials); these reagents were all purchased from Shanghai Macklin Biochemical Ltd. (Shanghai, China) and were analytical-grade reagents. The NR assay kit and nitrite assay kit were purchased from Nanjing Jiancheng Bioengineering Institute (Nanjing, China). All of these reagents and materials were dissolved in or diluted with ultrapure water with a resistivity greater than 18.2 MΩ·cm.

### 2.2. Experimental Procedure

The microalgae were precultured in an illumination incubator under the following conditions: luminance of 3000 lux, temperature of 25 °C, and 12 h light/dark cycles. When the density of the precultured microalgae reached 10^7^ cells/mL, the microalgae strain was employed in the batch experiment.

The batch experiment design is shown in Table S2. According to our previous study, the initial ROX concentrations for microalgae exposure were set at 0 (control), 0.1, 0.25, and 1 mg/L. Double-strength BG11, a 10 mg/L ROX stock solution, sterile water, and the prepared microalgae strain were sequentially added to 250 mL sterile conical flasks. All the treatments were set up in triplicate, and samples were collected at 0, 3, 7, 10, 14, and 21 d for the determination of growth, chlorophyll levels, nitrate reductase activity, nitrate and nitrite concentrations; the samples at each time point were set up independently.

### 2.3. Determination of Microalgal Cell Density and Chlorophyll Content

According to previous studies [28,32], the cell density of *C. pyrenoidosa* was determined using a combined method of microscopic counting and absorbance measurements. The cell density was calculated based on the fitted cell density versus absorbance correlation curve. The growth inhibition rate was calculated as follows:

μ = (C − T)/C × 100%(1)

where μ is the growth inhibition rate and C and T are the microalgae cell densities of the control and treated groups at 3, 7, 10, 14, and 21 d, respectively.

The chlorophyll-a (Chl-a), chlorophyll-b (Chl-b), and total chlorophyl contents were determined according to the method of a previous study with some improvements [31]. Briefly, 5 mL of the microalgae suspension was centrifuged, the supernatant was discarded, and the microalgae pellet was resuspended in 5 mL of 95% ethanol. Then, the sample was kept in a low-temperature environment with no light for 24 h, and then centrifuged again to obtain the supernatant that contained the chlorophyll. A_649_ and A_665_ are the absorbances of the supernatant at 649 and 665 nm, respectively. The chlorophyll a and b and total chlorophyl contents were calculated as follows:

Chl-a = 13.7 × A_665_ − 5.76 × A_649_ (2)

Chl-b = 25.8 × A_649_ − 7.6 × A_665_ (3)

Total chlorophyll = 20.04 × A_649_ + 6.1 × A_665_ (4)

### 2.4. Determination of NR Activity

A pretreatment was required before using the assay kit to determine the NR activity of the microalgae cells. Specifically, 30 mL of microalgae was centrifuged at 8000 rpm and washed several times using PBS buffer (pH = 7.2) to remove the environmental bacteria; we previously confirmed that the insignificant amount of remaining bacteria after this wash has no effect on the final NR activity [33]. The purified microalgae pellet without bacteria was ultrasonically lysed in 30 mL of phosphate buffer, and then the samples were subsequently centrifuged again to obtain the supernatants (cell lysates). The cell lysates containing NR were used to determine the NR activity; the determination procedure was performed according to the instructions of the assay kit [28].

### 2.5. Determination of Nitrate and Nitrite Concentrations

The nitrate concentration was determined using UV spectrophotometry. Briefly, 5 mL of microalgae was centrifuged, and the obtained supernatant was filtered to remove any residual particulate matter. The absorbances of the samples were measured at 220 and 275 nm after eliminating any interference. Based on the fitting curve for the concentration vs. absorbance graph of standard samples, the nitrate concentrations in the samples were calculated [34]. The filtered supernatant was also used for the determination of the nitrite concentration. The determination procedure was carried out in accordance with the instructions; the nitrite concentration was measured and calculated using UV–visible spectrophotometry at a wavelength of 550 nm [28].

### 2.6. Composition Analysis and Function Prediction of Environmental Bacteria

In the open and non-sterile ROX exposure system, the environmental bacteria rapidly colonized the surface of the microalgae cells. In order to analyze the changes in species composition and diversity of the environmental bacteria, the samples were centrifuged and the microalgae were collected and then frozen with liquid nitrogen. The composition and diversity of the environmental bacteria were determined by Majorbio Biotechnology Company (Shanghai, China). The experimental details and are described in Text S1 (Supplementary Materials), and the raw high-throughput sequencing data for the environmental bacteria was uploaded to the NCBI database under the accession number PRJNA1269455. Composition analysis and function prediction based on the sequencing data were performed and graphed using the Majorbio Cloud Platform (www.majorbio.com (accessed on 6 December 2022)).

### 2.7. Statistical Analysis

All biological parameters and concentrations are presented as the mean ± standard deviation from triplicate measurements. The significance level between the control and treatment groups was analyzed by one-way analysis of variance based on the Least Significant Difference (LSD) using SPSS 18.0. The figures were generated using Origin 2018.

## 3. Results and Discussion

### 3.1. Growth Changes of Microalgae Under ROX Exposure

**Figure 1:**
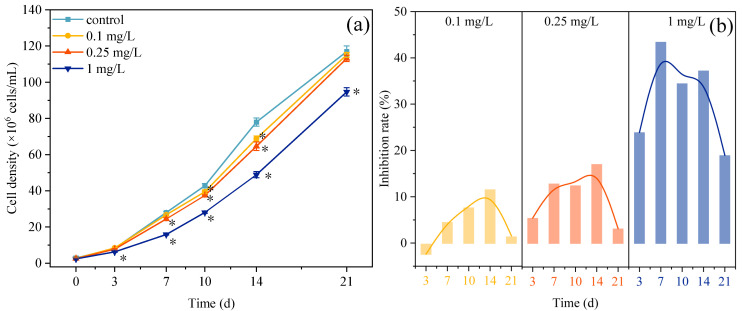
Dynamics of cell density (**a**) and inhibition rate (**b**) of *C. pyrenoidosa* exposed to varying ROX concentrations (0.1, 0.25, and 1 mg/L). * represents significant differences between the treatment group and the control group.

Throughout the 21-day exposure to 0.1, 0.25, and 1 mg/L ROX, the growth of the microalgae was consistently inhibited, except for a transient increase with the 0.1 mg/L treatment on day 3 (Figure 1a). For example, the microalgal cell densities in the control group were 8.21, 28.01, 42.80, 77.94, and 116.93 (×10^6^ cells/mL) on days 3, 7, 10, 14, and 21, respectively. The microalgal cell densities in the 0.25 mg/L treatment group were 7.76, 24.39, 37.43, 64.59, and 113.12 (×10^6^ cells/mL) at the same time points. The cell density in the treatment groups remained consistently lower than that of the control group, with the 1 mg/L treatment group exhibiting the most pronounced reduction. However, the inhibition rate in the treatment groups was not constant over the 21-day exposure (Figure 1b). In the early stages of ROX exposure, an increasing growth inhibition effect was observed at all concentrations. The inhibition rates in the 0.1 and 0.25 mg/L treatment groups gradually increased between 3 and 14 d, reaching peak values of 11.62% and 17.14%, respectively, followed by a gradual decline after 14 d. However, the inhibition rate in the 1 mg/L treatment group reached the highest value of 43.43% on day 7 and subsequently declined gradually. These results were consistent with many previous studies that showed that ROX exposure inhibits the growth of microalgae [16,35,36]. The observed growth inhibition may be attributed to ROX-induced interference with photosynthetic pigment biosynthesis [17], impaired organelle functions resulting from lipid peroxidation of cell membranes [35], the suppression of xenobiotic metabolism-related gene expression [36], and the inhibition of nitrogen metabolism-associated gene expression [16]. However, in the present study, we observed a consistent decline in the inhibition rates in all three treatment groups, although the reduction occurred at different time points. For instance, it decreased between 14 and 21 d in the 0.1 and 0.25 mg/L treatment groups and between 7 and 21 d in the 1 mg/L treatment group. Previous studies have suggested that the self-defense mechanism of microalgae can mitigate the growth inhibition induced by antibiotics. On the one hand, antioxidant enzymes, such as superoxide dismutase, catalase, and glutathione peroxidase, scavenge reactive oxygen species to alleviate oxidative damage from antibiotics [37]. On the other hand, microalgae adaptively modulate the composition of various photosynthetic pigments to compensate for the reduced light conversion efficiency caused by antibiotic-induced photosystem damage [38]. In addition, previous studies demonstrated that antibiotics can be effectively removed and degraded by microalgae systems through photolytic and biological processes. These processes collectively contribute to the attenuation of the antibiotic concentration through bio-adsorption, accumulation, and the transformation of the highly toxic antibiotic into less toxic degradation products [39,40]. Many studies have also documented the growth-promoting effect of symbiotic microorganisms on microalgae [41,42], but this enhanced resistance to antibiotics remains poorly characterized.

### 3.2. Chlorophyll Contents of Microalgae Under ROX Exposure

**Figure 2:**
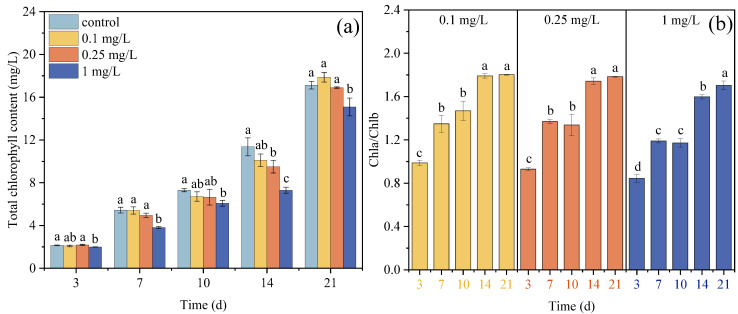
Effects of ROX exposure (0.1, 0.25, and 1 mg/L) on the chlorophyll content and Chl-a/Chl-b ratio of *C. pyrenoidosa*. Different letters represent the significance of the differences between the groups.

The Chl contents in the microalgae cells showed different responses to 0.1, 0.25, and 1 mg/L ROX exposure (Figure 2a). In the 0.1 mg/L treatment group, the total chlorophyll content showed no significant changes compared with the control during the initial 14 days, but exceeded the control level on day 21, though it was not statistically significant. The total chlorophyll content in the 0.25 mg/L treatment group was consistently slightly lower than that of the control group, with no significant differences. However, compared to the control group, the total chlorophyll content in the 1 mg/L treatment group was significantly lower throughout the 21-day exposure (*p* < 0.05). These demonstrated that a lower concentration of ROX has no significant impact on the total chlorophyll synthesis in microalgae, whereas higher concentrations of ROX have a significant inhibition effect on the total chlorophyll content. Many studies have indicated that the self-defense mechanism of microalgae can maintain physiological homeostasis under lower concentrations of antibiotics, but this mechanism is insufficient to compensate for the toxic damage caused by antibiotic stress at higher concentrations [37,43]. It has been increasing observed that under the hormesis effect of photosynthesis pigments in microalgae cells under antibiotic exposure, the mobilization of the antioxidant system, extracellular polymeric substances, and photosynthesis system play key roles [44,45]. Adaptive regulation of the photosynthesis system was confirmed in the dynamic adjustments to the pigment composition. The Chl-a/Chl-b ratio serves as a more sensitive indicator of photosynthetic efficiency compared to the total chlorophyll content and provides valuable insights into microalgae responses to environmental stress [41]. In this study, the Chl-a/Chl-b ratios in all treatment groups increased between 3 and 21 d (Figure 2b). This revealed that the microalgae adaptively modulated their chlorophyll composition by upregulating Chl-a levels and downregulating Chl-b levels, thereby enhancing the light energy conversion capacity to mitigate ROX-induced growth inhibition [46,47] because the smaller antenna size of PSII helps increase the photochemical efficiency through reducing the transferred excitation energy from PSII to PSI [48,49]. These results provide a reasonable explanation for the reduced growth inhibition in the three treatment groups.

### 3.3. NR Activity of Microalgae Under ROX Exposure

**Figure 3:**
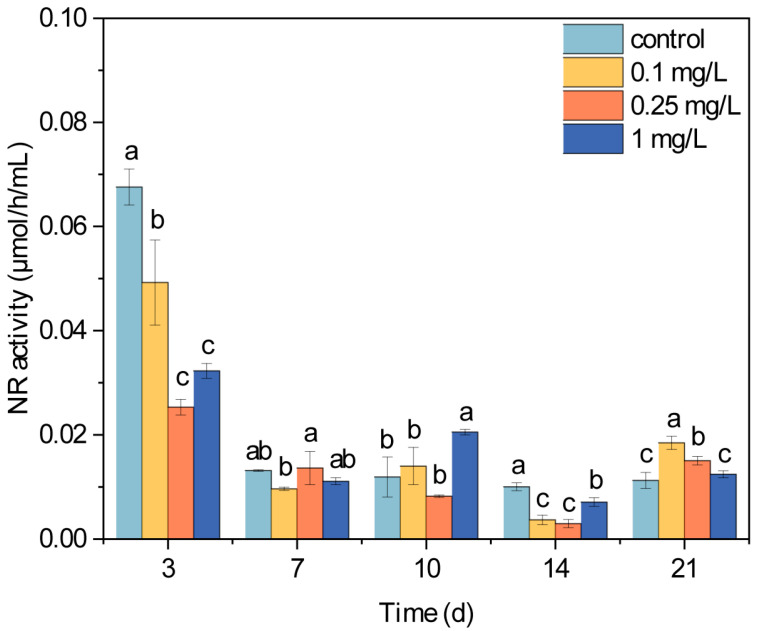
Effects of ROX exposure (0.1, 0.25, and 1 mg/L) on the nitrate reductase activity of *C. pyrenoidosa*. Different letters represent the significance of the differences between the groups.

Antibiotic exposure disrupts the activity of various metabolic enzymes, consequently affecting microalgae growth. Previous studies observed antibiotic-induced alterations in the activity of enzymes involved in antioxidant defenses, carbon metabolism, and the photosynthetic pathways [22,50]. NR serves as a key catalyzing and transferring enzyme in the nitrogen metabolism of microalgae cells, which mediates the sequential reduction of nitrate to nitrite and ultimately to ammonium, the essential precursor for amino acid biosynthesis [51]. Beyond photosynthetic limitations on microalgae growth, nitrogen metabolism efficiency also influences the effects on microalgae growth under antibiotic stress [20]. The NR activity in the microalgae cells exhibited significant dynamic changes throughout the 21-day ROX exposure period (Figure 3). After 3 d of ROX exposure, the NR activities in all the treatment groups significantly decreased compared to the control group (*p* < 0.05). There were no significant differences between the control and treatment groups on day 7, and only the 1 mg/L treatment group showed a significantly higher level than that of the control group on day 10. After 14 d of ROX exposure, all the treatment groups exhibited significantly lower NR activities compared to the control group, and the 1 mg/L treatment group showed higher activity than the 0.1 and 0.25 mg/L treatment groups. However, after 21 d, all the treatment groups exhibited higher NR activities than the control group, with the two lower concentration groups showing statistically significant increases. Overall, the NR activity of the microalgae exhibited a biphasic response to ROX exposure, that is to say, the NR activity was initially suppressed during the early stage of ROX exposure, but progressively recovered to the control level and even exceeded the control level in the later phase. These results correspond with the beforementioned decrease in inhibition rates and increase in Chl-a/Chl-b ratios, which suggest coordinated physiological adaption. Previous studies have similarly documented, under sublethal antibiotic exposure, the stimulation of the activity of microalgae enzymes such as superoxide dismutase [52], catalase [53], cytochrome P450 [54], and carbonic anhydrase [55]. These stimulated enzyme activities collectively maintain cellular metabolic homeostasis, thereby restoring the microalgae growth capacity under antibiotic stress. In this study, the increased NR activity observed during the later ROX exposure stage facilitated nitrate uptake and biotransformation in the microalgae cells, thereby establishing an essential metabolic foundation for microalgae growth recovery.

### 3.4. Concentrations of Nitrate and Nitrite in Exposure System

**Figure 4:**
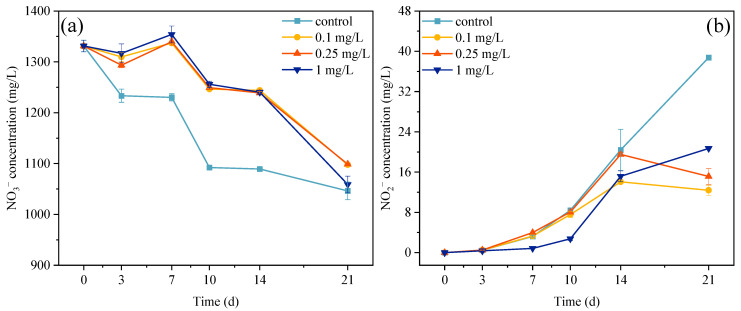
Nitrate consumption (**a**) and nitrite production (**b**) in the exposure system containing different concentrations of ROX.

Nitrogen metabolism is closely related to microalgae growth, and the nitrification and denitrification processes mediated by microalgae–bacteria consortia drive nitrogen speciation changes that are critical for microalgae growth [56,57]. In this study, microalgae–bacteria consortia were cultured in an artificial culture medium containing NaNO_3_ and Co(NO_3_)_2_·6H_2_O; thus, the initial species of nitrogen was nitrate with a concentration 1331.22 mg/L (Figure 4a). During the entire 21-day exposure period, the nitrate concentrations in three ROX treatment groups were all higher than that of the control group. These results indicated that ROX reduced the nitrate consumption rates of the microalgae, and this was consistent with the microalgae growth results. In addition, in the first 14 d of ROX exposure, there were significant differences in the nitrate consumption between the treatment groups and the control group, but the difference decreased at 21 d. These results are also consistent with the decreased growth inhibition rates of the microalgae. In terms of nitrite, the presence of denitrifying bacteria led to a progressive increase in the nitrite concentration within the exposure system in the control and treatment groups (Figure 4b). However, in the first 14 d, the nitrite concentrations in the three treatment groups were always lower than the control group. For example, the 0.1 mg/L treatment group showed nitrite concentrations of 108.14%, 102.34%, 89.99%, 68.94%, and 31.98% relative to the control group at 3, 7, 10, 14, and 21 d, respectively. After 14 d, the nitrite concentration in the control group maintained its rapid growth, while all the treatment groups showed either slowed increased or decreasing concentrations. These results demonstrated that ROX not only inhibited microalgae growth, but may have also affected the functions of specific bacteria populations. In the early stage of ROX exposure, the NR activity in the microalgae cells was inhibited by ROX exposure, consequently decelerating the nitrate metabolic rate in the microalgae cells and ultimately inhibiting microalgae growth [58]. But, in the later stage, the NR activity in the microalgae cells returned to normal levels, resulting in a rapid decrease in the nitrate concentration in the treatment groups [58,59]. On the other hand, the process of converting nitrate into nitrite also influenced the consumption rate of nitrate within the exposure system. However, this environmental bacteria-mediated conversion process had limited efficacy, and the resulting nitrite accumulation could still potentially impact microalgae growth. Previous studies indicated that the nitrite concentration could affect the growth of microalgae, and a 16–66 mg/L nitrite concentration was observed to inhibit the growth of microalgae through impairing the PsbO subunit in photosystem II [60,61]. In this study, the nitrite concentration increased with exposure time, but the treatment groups showed lower nitrite concentrations compared to the control group; thus, ROX seemed to alleviate the stress from high nitrite concentrations.

### 3.5. Changes in the Composition and Diversity of Environmental Bacteria

**Figure 5:**
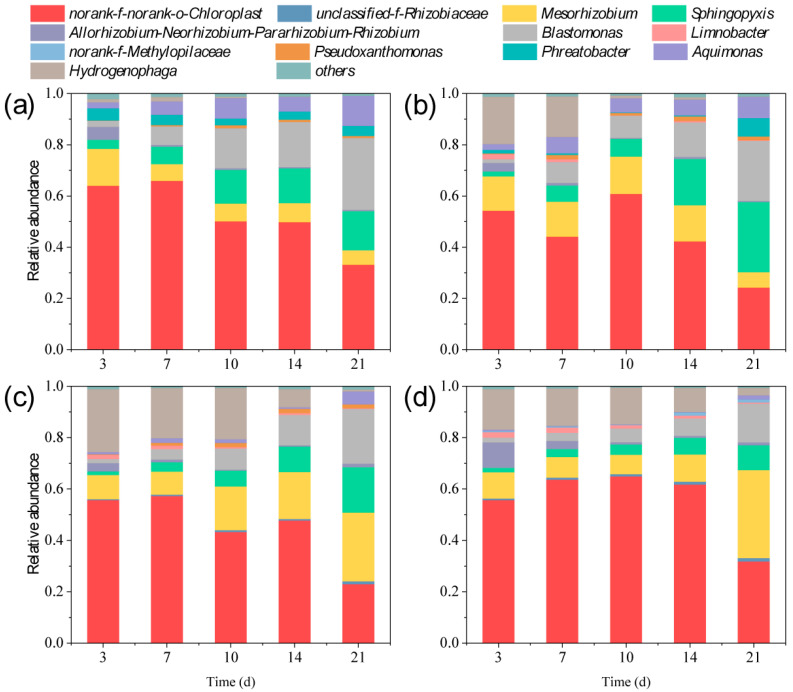
Effects of control (**a**), 0.1 (**b**), 0.25 (**c**), and 1 (**d**) mg/L ROX on the community composition of environmental bacteria at the genus level.

To investigate the effects of the environmental bacteria on nitrogen metabolism and transformation in the exposure system, this study successively examined both the compositional and functional changes in the environmental bacteria. Therefore, the environmental bacteria were harvested for DNA extraction, and the DNA was amplified and sequenced. The sequencing results indicated that 3,542,746 gene sequences were detected in the 60 samples. The mean, maximum, and minimum lengths of the sequences were 409, 517, and 201, respectively. A total of 12 abundance values had a relative abundance greater than 1% (Figure 5), among which was *norank-f-norank-o-Chloroplast*, which are commonly detected gene sequences from chloroplasts. Sequences belonging to this category are usually detected in communities of photosynthetic microorganism [62,63]. At the genus level, 11 bacterial genera were detected in the samples from day 21 of ROX exposure, and the antibiotic had obvious effects on the community composition of the environmental bacteria. For instance, *unclassified-f-Rhizobiaceae* was absent in both the control group and 0.1 mg/L treatment group (relative abundance < 1%); however, it was observed in the 0.25 and 1 mg/L treatment groups. The relative abundance of *Mesorhizobium* decreased from 14.41% to 5.56% and from 13.39% to 6.04% in the control and 0.1 mg/L treatment groups, but increased from 9.38% to 26.73% and from 10.16% to 34.22% in the 0.25 and 1 mg/L treatment groups. The relative abundance of *Sphingopyxis*, *Blastomonas*, and *Aquimonas* increased in all groups. The relative abundance of *Hydrogenophaga* decreased in all groups. *Limnobacter* was detected in the ROX treatment groups but not in the control group (relative abundance < 1%). *Norank-f-Methylopilaceae* was only observed in the 1 mg/L treatment group. *Pseudoxanthomonas* and *Phreatobacter* were observed in the control and 0.1 mg/L treatment groups but not in the 0.25 and 1 mg/L treatment groups (relative abundance < 1%).

Alpha diversity analysis at the genus level of the environmental bacteria was performed using complementary metrics; the Chao1 and Ace indices were used to assess community richness; and the Simpson and Shannon indices were used to evaluate community diversity (Figure S1, Supplementary Materials). The Chao1 and Ace indices of the treatment groups were lower than that of the control group at 3 d, with significant differences in the Chao1 index. After 3 d, compared with the control group, the Chao1 and Ace indices in the treatment groups exhibited fluctuating increases with a gradual decrease in the differences. During the initial ROX exposure period, no statistically significant differences were observed in the Simpson and Shannon indices between the treatment groups and the control group. But, by 21 d, the Simpson index exhibited a ROX concentration-dependent increase, while the Shannon index showed an inverse relationship. There were significant differences between the 0.1 and 1 mg/L treatment groups for both indices.

The primary mechanism of ROX is the inhibition of protein biosynthesis in microbial cells, which not only affects microalgae growth, but also the composition of the bacterial community [64]. In this study, the environmental bacteria were obviously affected during the 21 d of ROX exposure. The changes in the community composition inevitably altered the relationship between the environmental bacteria and microalgae. *Unclassified-f-Rhizobiaceae* and *Mesorhizobium* are important nitrogen-fixing bacteria in microalgae–bacteria consortia [65,66]; the former was only detected in the higher ROX concentration treatment groups, and the relative abundance of the latter increased in the higher ROX concentration treatment group. These results indicate that ROX exposure may enhance the nitrogen fixation and utilization in microalgae–bacteria consortia and that microalgae rely on this strategy to resist the toxic stress caused by higher ROX concentrations [67]. In addition, *Limnobacter*, *Sphingopyxis*, and *Aquimonas* appeared or increased in the ROX treatment group with exposure time. Previous studies suggested that these bacterial genera are related to the biodegradation of organic pollutants as they can produce various degrading enzymes to accelerate the degradation of antibiotics or mediate microalgae cells to biosynthesize metabolites to enhance their resistance to antibiotics [68,69]. Overall, compared with the initial stage, ROX exposure slightly increased the richness and stabilized the diversity of the environmental bacteria. These findings indicate that microalgae and bacteria jointly create a stable symbiotic system to resist antibiotic stress.

### 3.6. Metabolic and Ecological Functions of Environmental Bacteria

**Figure 6:**
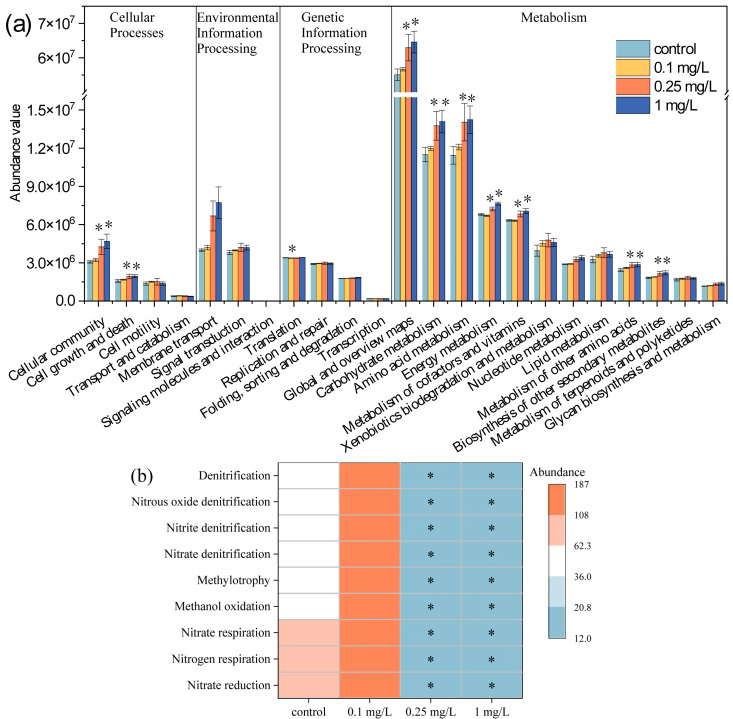
Metabolic (**a**) and ecological (**b**) functions of environmental bacteria predicted by PICRUSt2 and FAPROTAX at 21 d. * represents significant differences between the treatment group and the control group.

To comprehensively evaluate the functional role of the environmental bacteria in the microalgae–bacteria consortia, we predicted the metabolic and ecological functions of the environmental bacteria based on 16S rRNA sequencing. Figure 6a shows the changes in the metabolic functions of the environmental bacteria predicted by PICRUSt2, a bioinformatic tool designed to predict bacterial functions from 16S rRNA data using an annotated genome database. A total of 23 functional pathways at level 2 were detected; these pathways are related to cellular processes, environmental information processing, genetic information processing, and metabolism. In this study, compared with the control group, the 0.25 and 1 mg/L ROX treatments significantly increased (*p* < 0.05) the activity of metabolic pathways involved in cellular communities (38.86–52.86%), cell growth and death (22.42–24.10%), global and overview maps (14.33–17.30%), carbohydrate metabolism (19.71–22.61%), amino acid metabolism (22.79–24.50%), energy metabolism (6.31–12.52%), metabolism of cofactors and vitamins (7.83–11.32%), metabolism of other amino acids (16.72–17.68%), and biosynthesis of other secondary metabolites (17.32–20.97%) were significantly increased by 0.25 and 1 mg/L ROX (*p* < 0.05). The prediction results indicated that higher ROX concentration enhanced above metabolic pathways in the environmental bacteria. Previous studies found that ciprofloxacin enhanced the metabolic functions of microbial communities, including carbohydrate metabolism, the metabolism of cofactors and vitamins, and energy metabolism [70]. Erythromycin and telithromycin have also been observed to upregulated the metabolism of cofactors and vitamins in bacterial communities [71]. Metabolites produced by environmental bacteria, such as cofactors and vitamins, enhance microalgae growth and resistance to environmental stress. For example, vitamin B12 synthesized by environmental bacteria participates in methionine biosynthesis in microalgae, thereby regulating microalgae growth, while vitamin B1 indirectly modulates photosynthetic efficiency [72,73]. Additionally, coenzyme Q plays a pivotal role in reactive oxygen species scavenging and stress resistance [74,75].

Figure 6b shows the changes in the ecological functions of the environmental bacteria predicted by FAPROTAX. The prediction results indicated that the nitrogen metabolism processes were significantly affected by ROX. The nitrogen metabolism processes encompass several distinct but related mechanisms. Firstly, there is nitrogen respiration, an anaerobic respiratory process wherein bacteria employ nitrate as the terminal electron acceptor to produce metabolic energy. Secondly is nitrogen denitrification, a nitrogen transformation process mediated by denitrifying bacteria during which, various nitrogen species are progressively reduced to gaseous nitrogen. Lastly is nitrogen reduction, a metabolic process that comprises both the dissimilatory and assimilatory reduction pathways for nitrogen [76,77]. In this study, the 0.25 and 1 mg/L treatments significantly inhibited the above three processes of nitrogen metabolism (*p* < 0.05), while no significant difference was observed between the 0.1 mg/L treatment group and the control group (*p* > 0.05). Previous studies also reported that the nitrogen metabolism of bacterial communities can be suppressed by high levels of metal and antibiotic stress, and that key denitrification genes such as *nirK*, *nirS*, and *nosZ* may be downregulated in toxic stress [78,79]. The primary nitrogen form in the exposure system was nitrate. The observed inhibition of denitrification functions reduced the conversion of nitrate to biologically unavailable forms, which is consistent with the nitrite accumulation results. Sufficient nitrate helps facilitate nitrogen assimilation and the growth of microalgae cells.

### 3.7. Correlation Between Microalgae and Environmental Bacteria

**Figure 7:**
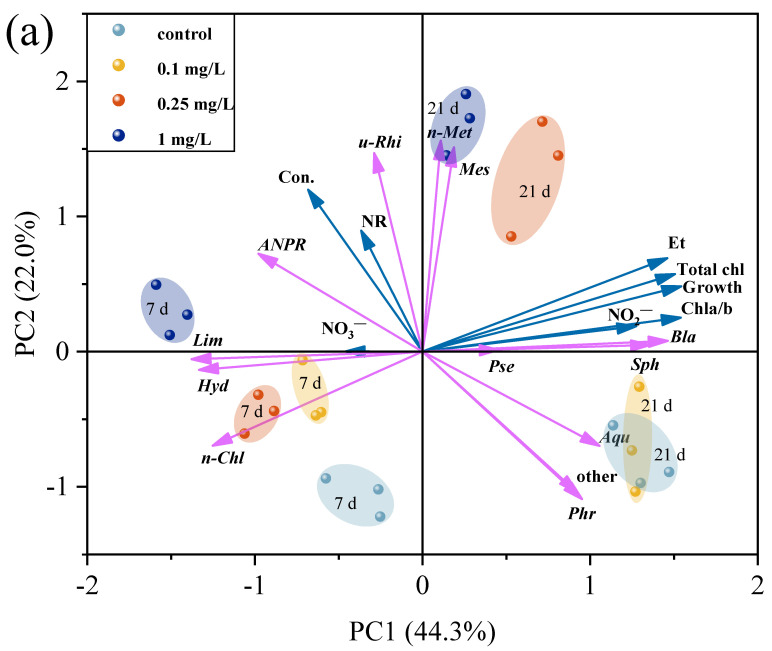

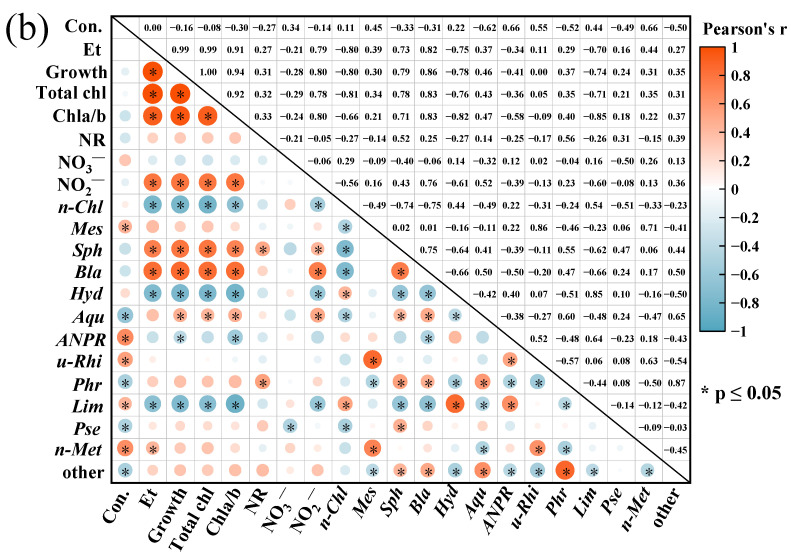
Principal component analysis of microalgal parameters and bacterial abundances (**a**). Pearson’s correlation analysis of microalgal parameters and bacterial abundances (**b**). Abbreviations: Con., concentration of ROX; Et, exposure time; Growth, microalgae growth; Total chl, total chlorophyll content of microalgae; Chl-a/b, chlorophyll a/chlorophyll b ratio; NR, nitrate reductase activity; NO_3_^−^, nitrate concentration; NO_2_^−^, nitrite concentration; *n-Chl*, *norank-f-norank-o-Chloroplast*; *Mes*, *Mesorhizobium*; *Sph*, *Sphingopyxis*; *Bla*, *Blastomonas*; *Hyd*, *Hydrogenophaga*; *Aqu*, *Aquimonas*; *ANPR*, *Allorhizobium-Neorhizobium-Pararhizobium-Rhizobium*; *u-Rhi*, *unclassified-f-Rhizobiaceae*; *Phr*, *Phreatobacter*; *Lim*, *Limnobacter*; *Pse*, *Pseudoxanthomonas*; *n-Met*, *norank-f-Methylopilaceae*. The lengths of the arrows indicate the variances of the data points in the corresponding principal component direction. The size of the circle represents the absolute value of Pearson’s r.

To better understand the relationship between the microalgae and environmental bacteria and clarify the effects of the environmental bacteria on microalgae metabolism, PCA was performed (Figure 7a). The Principal Component Analysis (PCA) results showed apparent clusters between the control, 0.1, 0.25, and 1 mg/L treatment groups on days 7 and 21. The two axes explained 66.3% of the total variance, indicating that the data was suitable for this analysis. The PC1 axis explained 44.3% of the total variance, and it was beneficial for distinguishing the effects of ROX exposure time on the microalgae and environmental bacteria. PC1 was mainly affected by the total chlorophyll content, growth, and Chl-a/b ratio of the microalgae, the nitrate and nitrite concentrations of exposure system, the abundance of *Blastomonas*, *Sphingopyxis*, *Limnobacter*, and *Hydrogenophaga*. The PC2 axis accounted for 22.0% of the total variance and can effectively discriminate between the different exposure concentrations. It is worth noting that the 0.1 mg/L treatment group overlapped with the control group on day 21; this was related to the fact that there was no significant difference in the various indicators between the control group and 0.1 mg/L treatment group, including growth, chlorophyll content, and the composition and function of the bacterial community. PC2 was mainly affected by the nitrate reductase activity of the microalgae and the abundance of *unclassified-f-Rhizobiaceae*, *norank-f-Methylopilaceae*, *Mesorhizobium*, and *Phreatobacter*.

Pearson’s correlation analysis was also conducted to describe the correlation between the microalgal indicators and bacterial abundances (Figure 7b). The abundances of *Mesorhizobium*, *Allorhizobium-Neorhizobium-Pararhizobium-Rhizobium*, *unclassified-f-Rhizobiaceae*, *Limnobacter*, and *norank-f-Methylopilaceae* were positively correlated with ROX concentration, but the abundances of *Aquimonas*, *Phreatobacter*, and *Pseudoxanthomonas* were negatively correlated with ROX concentration. The other bacteria showed a weak correlation, indicating that the environmental bacteria showed different sensitivities to ROX. The potential hosts of antibiotic resistance genes, such as *Mesorhizobium* and *Limnobacter*, typically exhibit enhanced tolerance to antibiotic exposure [80,81]. Meanwhile, the abundances of *Sphingopyxis*, *Blastomonas*, and *norank-f-Methylopilaceae* were significantly positively correlated with the ROX exposure time (*p* < 0.05) and the abundances of *Hydrogenophaga* and *Limnobacter* were significantly negatively correlated with the ROX exposure time (*p* > 0.05), indicating these bacterial abundances were strongly time-dependent. This observed time dependence may be related to the growth of the microalgae: as the microalgae density increased, the environmental bacteria and microalgae established a more stable symbiotic relationship [26]. Microalgal biomarkers like growth, total chlorophyll content, and Chla/b ratio were significantly positively correlated with the abundances of *Sphingopyxis*, *Blastomonas*, and *Aquimonas*, but significantly negatively correlated with the abundances of *Hydrogenophaga*, *Allorhizobium-Neorhizobium-Pararhizobium-Rhizobium*, and *Limnobacter*. *Sphingopyxis* is believed to be capable of producing extracellular polysaccharides. These polysaccharides, together with the extracellular polymers of microalgae, form a protective barrier and provide a source of nutrients [82,83]. Microalgae and some environmental bacteria bidirectionally provide nutrients and infochemicals, resulting a simultaneous increase in the cell numbers of both. Conversely, The opposite effects can also occur [26,84]. In addition, *Sphingopyxis* and *Aquimonas* have been reported to have the ability to degrade triphenyl phosphate and sulfadiazine, respectively [69,85]. The bacteria that establish stable relationships with microalgae have been found to be capable of degrading organic pollutants in water environments, suggesting that environmental bacteria also contribute to microalgae growth by alleviating the stress from antibiotics. On the other hand, as the microalgae density increases, the abundances of some genera decrease due to a lack of stable interspecific environmental relationships between the microalgae and bacteria. This environmental relationship may be disrupted by stress from contaminants [86,87] or changes in extracellular polymeric substances [88]. For instance, *Limnobacter*, a denitrifying bacteria, converts nitrate into N_2_; it competes with the microalgae by absorbing and utilizing nitrate, ultimately leading to the growth of dominant microalgae cells while the bacterial abundance decreases [89].

## 4. Conclusions

In summary, ROX inhibited the growth and chlorophyll synthesis of *C. pyrenoidosa*, but the inhibition rates continuously declined during the middle to late stages of exposure. In addition to the previously recognized role of antioxidant enzymes in resisting antibiotic stress, microalgae can also adjust their pigment composition and NR activity in response to ROX stress. The increased proportion of Chl-a enabled the adsorption and conversion of more light energy, while the elevated NR activity enhanced the efficiency of nitrogen metabolism. These measures were all beneficial for resisting antibiotic stress and promoting microalgae growth. In addition, the microalgae specifically recruited and adjusted the composition and functions of the environmental bacteria in response to the antibiotic stress. The abundance of environmental bacteria capable of nitrogen fixation and organic contaminant degradation increased. The enhanced metabolism of the bacterial community, particularly in cofactor and vitamin metabolism, was beneficial for microalgae growth. The reduction in the denitrification function of the bacterial community helped ensure the availability of bioavailable nitrogen in the system. By exchanging nutrients and infochemicals, the microalgae and bacteria established a stable symbiotic relationship, thereby enhancing the resistance of the microalgae to the antibiotic. These findings provide new insights into the self-defense mechanisms of microalgae against antibiotics, and also provide a scientific basis for microalgae-based wastewater treatment.

## Data Availability

Data will be made available on request. The 16S sequencing raw data are available at https://dataview.ncbi.nlm.nih.gov/object/PRJNA1269455?reviewer=sevu8bnq355va5jomnjr9moj94 (accessed on 29 May 2025).

## Supplementary Materials

The following supporting information can be downloaded at: https://www.mdpi.com/article/10.3390/plants14172774/s1.
