# Supplementary material for: Effects of Roxithromycin Exposure on the Nitrogen Metabolism and Environmental Bacterial Recruitment of Chlorella pyrenoidosa
Source: Plants (Basel). 2025 Sep 4;14(17):2774. doi: 10.3390/plants14172774 (PMC12430757; doi:10.3390/plants14172774)
Supplement: Supplementary file 1 [file plants-14-02774-s001.zip › plants-3824399-supplementary.pdf]

Supplementary materials

for

Effects of roxithromycin exposure on the nitrogen metabolism and environmental bacterial recruitment of *Chlorella pyrenoidosa*

Jiping Li [a,b], Ying Wang [a,b], Zijie Xu [a], Chenyang Wu [a], Zixin Zhu [a], Xingsheng Lyu [a], Jingjing Li [a], Xingru Zhang [a], Yan Wang [a,b], Yuming Luo [a,b], Wei Li [c,*]

a. School of Life Sciences, Huaiyin Normal University, Huaian 223300, China
b. Jiangsu Collaborative Innovation Center of Regional Modern Agriculture & Environmental Protection, Huaiyin Normal University, Huaian 223300, China
c. College of Ecology and Environment, Nanjing Forestry University, Nanjing 210037, China
* Correspondence: College of Ecology and Environment, Nanjing Forestry University, Longpan Road 159, Nanjing 210037, China; E-mail address: uwliwei@163.com (Wei Li)

Table S1 Components of BG11 medium used for microalgae culture

Table S2 Experimental design and the volume (mL) of each component

Text S1 Composition analysis and function prediction of symbiotics bacteria

Fig S1 Alpha diversity analysis of environmental bacteria around microalgae under ROX exposure

Table S1 Components of BG11 medium used for microalgae culture

Component		Concentration of stock solution	Volume of stock solution in 1 L medium
$NaNO_3$		75 g/500 mL dH_2O	10 mL/L
K_2HPO_4		2g/500 mL dH_2O	10 mL/L
$MgSO_4·7H_2O$		3.75 g/500 mL dH_2O	10 mL/L
$CaCl_2·2H_2O$		1.8 g/500 mL dH_2O	10 mL/L
Citricacid		0.3 g/500 mL dH_2O	10 mL/L
Ferric ammonium citrate		0.3 g/500 mL dH_2O	10 mL/L
$EDTANa_2$		0.05 g/500 mL dH_2O	10 mL/L
Na_2CO_3		1.0 g/500 mL dH_2O	10 mL/L
A5	H_3BO_3	2.86 g/L dH_2O	1 mL/L
	$MnCl_2·4H_2O$	1.86 g/L dH_2O	
	$ZnSO_4·7H_2O$	0.22 g/L dH_2O	
	$Na_2MoO_4·2H_2O$	0.39 g/L dH_2O	
	$CuSO_4·5H_2O$	0.08 g/L dH_2O	
	$Co(NO_3)_2·6H_2O$	0.05 g/L dH_2O	

Adjust pH to 7.1 with 1 M NaOH or HCl

Table S2 Experimental design and the volume (mL) of each component

Treatments	BG11 of double-strength	10 mg/L ROX	Sterile water	Microalgae strain
Control	50	0	40	10
0.1 mg/L	50	1	39	10
0.25 mg/L	50	2.5	37.5	10
1 mg/L	50	10	30	10

Text S1 Composition analysis and function prediction of symbiotics bacteria

The analysis of phycospheric bacterial community was conducted by Majorbio Biotechnology Company (Shanghai, China). Briefly, bacterial genomic DNA was collected from the phycosphere of microalgae at 3, 7, 10, 14, and 21 days, followed by extraction using the E.Z.N.A.® Soil DNA Kit (Omega Bio-tek, Norcross, GA, USA). The extracted DNA was then electrophoresed on a 1% agarose gel, and its concentration and purity were determined using a NanoDrop 2000 UV-vis spectrophotometer (Thermo Fisher Scientific, Waltham, MA, USA). The V3 – V4 region of the bacterial 16S RNA gene was amplified using an ABI GeneAmp® 9700 PCR thermocycler (ABI, Foster City, CA, USA) with the primer pair 338F (5′-ACTCCTACGGGAGGCAGCAG-3′) and 806R (5′-GGACTACHVGGGTWTCTAAT-3′). Each sample was amplified in triplicate, and the PCR products were purified after extraction from a 2% agarose gel. The purified products were pooled and subjected to paired-end sequencing on an Illumina MiSeq PE300 platform (Illumina, San Diego, CA, USA).

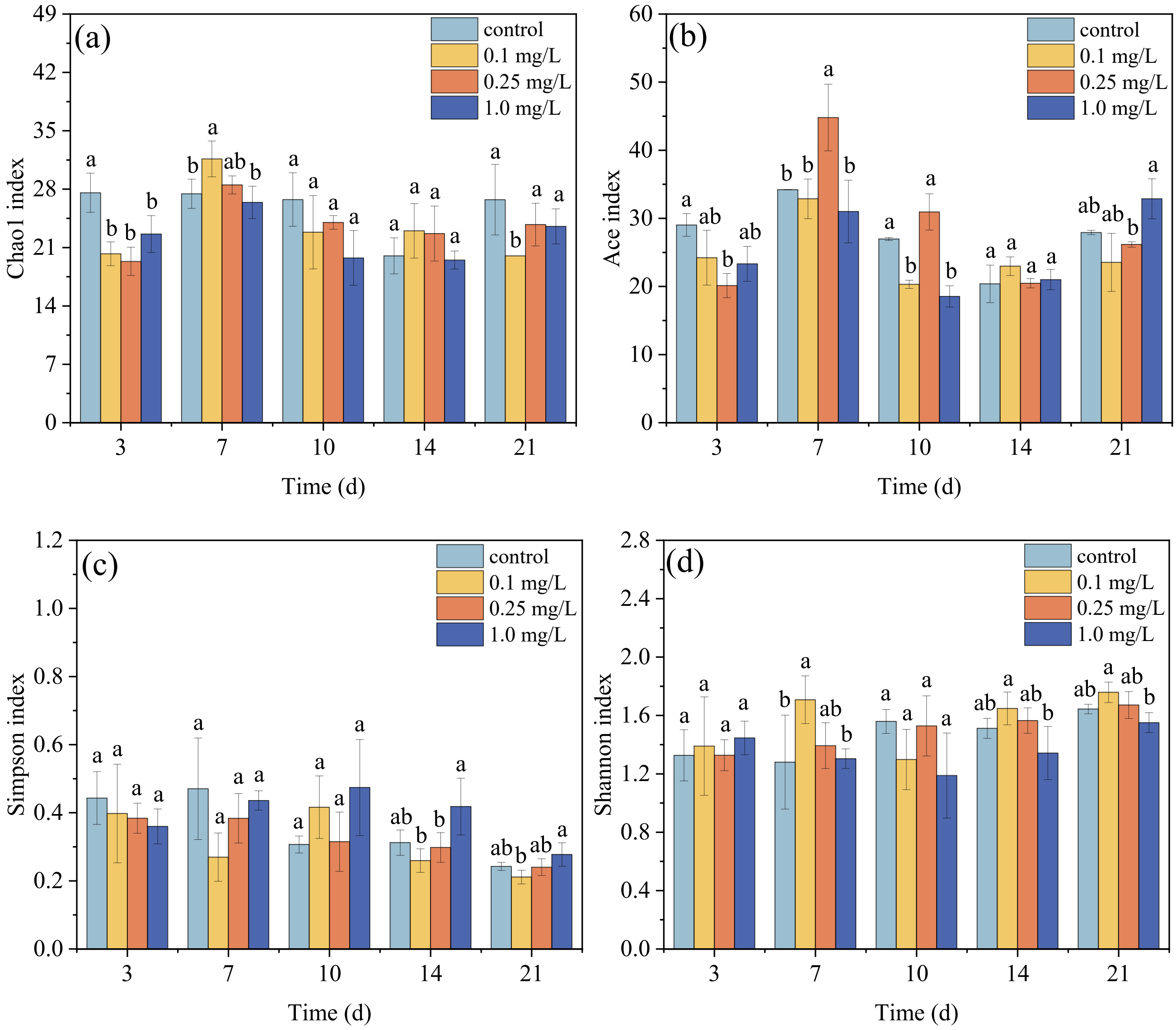

Fig S1 Alpha diversity analysis (Chao1 index (a), Ace index (b), Simpson index(c), Shannon index (d)) of environmental bacteria around microalgae under ROX exposure
